# LEGO-compatible modular mapping phantom for magnetic resonance imaging

**DOI:** 10.1038/s41598-020-71279-1

**Published:** 2020-09-08

**Authors:** Hyo-Min Cho, Cheolpyo Hong, Changwoo Lee, Huanjun Ding, Taeho Kim, Bongyoung Ahn

**Affiliations:** 1 Safety Measurement Institute, Korea Research Institute of Standards and Science (KRISS), Daejeon, 34113 Republic of Korea; 2 Department of Radiological Science, Daegu Catholic University, Gyeongsan-si, 38430 Gyeongbuk Republic of Korea; 3 Department of Radiological Sciences, University of California, Irvine, CA 92697 USA; 4 Department of Radiation Oncology, Washington University, Saint Louis, MO 63110 USA

**Keywords:** Biomarkers, Medical research

## Abstract

Physical phantoms have been widely used for performance evaluation of magnetic resonance imaging (MRI). Although there are many kinds of physical phantoms, most MRI phantoms use fixed configurations with specific sizes that may fit one or a few different types of radio frequency (RF) coils. Therefore, it has limitations for various image quality assessments of scanning areas. In this article, we report a novel design for a truly customizable MRI phantom called the LEGO-compatible Modular Mapping (MOMA) phantom, which not only serves as a general quality assurance phantom for a wide range of RF coils, but also a flexible calibration phantom for quantitative imaging. The MOMA phantom has a modular architecture which includes individual assessment functionality of the modules and LEGO-type assembly compatibility. We demonstrated the feasibility of the MOMA phantom for quantitative evaluation of image quality using customized module assembly compatible with head, breast, spine, knee, and body coil features. This unique approach allows comprehensive image quality evaluation with wide versatility. In addition, we provide detailed MOMA phantom development and imaging characteristics of the modules.

## Introduction

Image quality assessments are a key component for the validity of magnetic resonance imaging (MRI) system performance evaluations^1–3^. The objective assessments for image quality are performed through imaging a physically known object, a phantom, which has an artificially constructed representation^4–6^. The physical phantom with specially designed structures is employed to measure image quality parameters such as contrast resolution, spatial resolution, signal-to-noise ratio (SNR), uniformity, slice thickness, and geometric distortion^7,8^.

Various physical phantoms from phantom manufacturers, imaging system vendors, and international organizations are shown in supplementary Fig. 1a. The American College of Radiology (ACR) accreditation phantom was introduced in 1992 and has been widely used in image quality evaluation^1,4,9,10^. Following the ACR MRI quality control manual^11^, this phantom can be used to evaluate various image quality metrics, including geometry accuracy, high-contrast spatial resolution, low-contrast detectability, slice position accuracy, and slice thickness accuracy, using a specific set of protocols. The 76-904 MRI surface coil phantom was designed for comprehensive assessment of surface coils, but this phantom has been discontinued^12^. The Magphan quantitative imaging phantom was introduced in 2010 and includes detailed measurements of geometric distortion in MRI systems^6,13,14^. This phantom has been widely used for distortion monitoring in neuroimaging studies. With the increasing use of MRI systems for therapy planning and multimodal imaging, the CIRS model 603A and triple modality 3D abdominal phantom have been used to quantify the MR image distortion for stereotactic radiosurgery Planning (SRS) and multimodal imaging system validation ^15,16^. As shown in supplementary Fig. 1a, the physical phantoms are becoming more diverse with the increased development of MRI technology.

**Figure 1:**
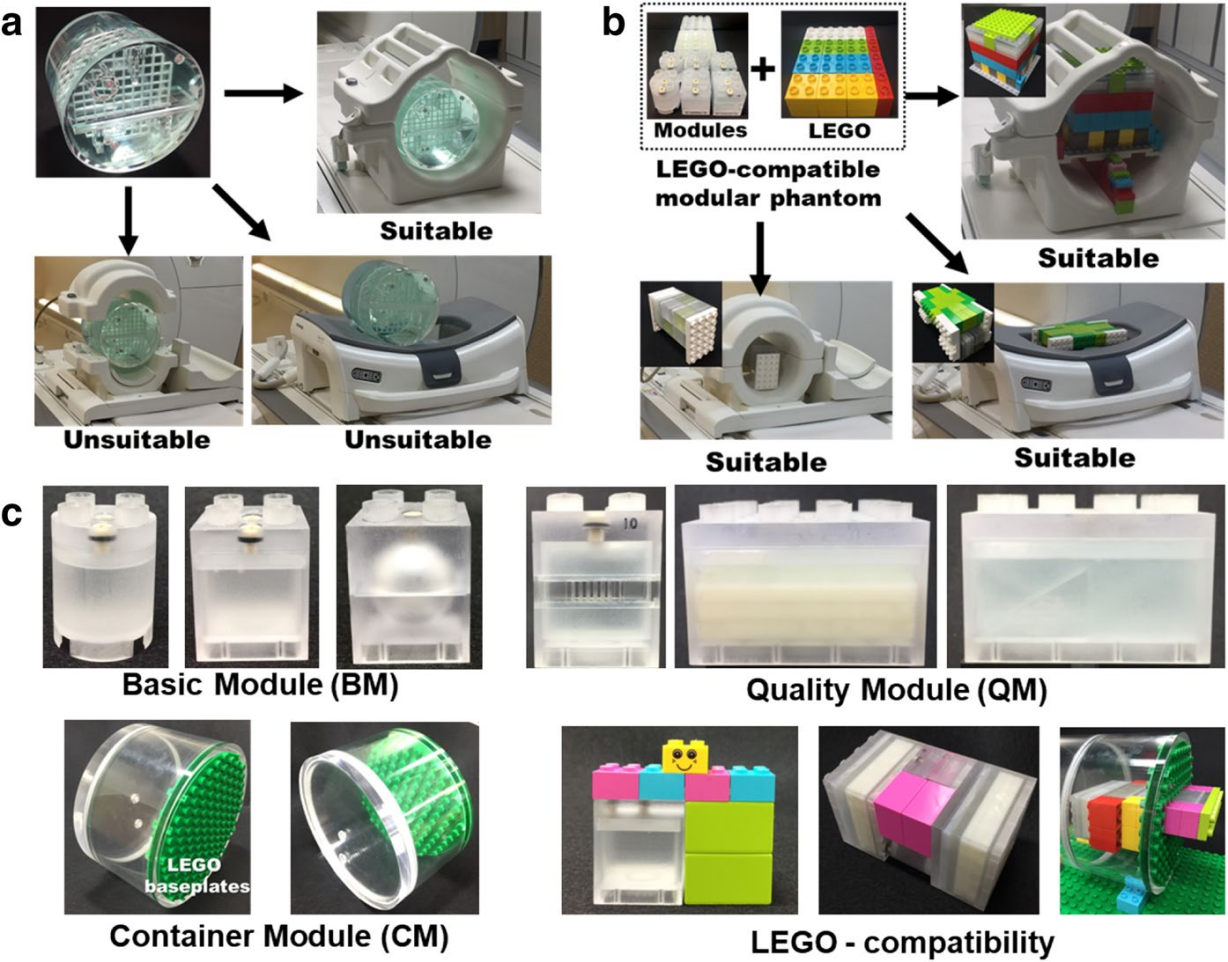
LEGO-compatible MOMA phantom. **(a)** The ACR MRI accreditation phantom fits into the head imaging coil but cannot be used with the breast and knee coil. **(b)** LEGO-compatible MOMA phantom is well-suited for head, breast, and knee coil. **(c)** LEGO-compatible MOMA phantom is composed of BM, QM, and CM. A developed phantom of any size and configuration can be constructed by dividing or multiplying the modules. LEGO-compatible connection enables versatile combinations of the BM, QM, and CM. Also, LEGO bricks can be used for a jig, fixture, and cradle.

MRI uses many different types and channels of radio frequency (RF) coils, such as surface coils, volume coils, and array coils with parallel-, phased-, and decoupled-types, to optimize the transmission and the reception of the MR signal^17,18^. These coils vary significantly in terms of physical dimensions and technical specifications to accommodate various clinical applications for different anatomical regions, such as the head, breast, body, knee, wrist, and shoulder (Supplementary Fig. 1b). Moreover, the configuration of RF coils varies across manufacturers. Commonly used MRI phantoms are typically fixed in size and configuration, and therefore they can only be used with one or a few types of RF coils. For instance, the ACR MRI accreditation phantom, which has an inside diameter of 190 mm, was fabricated to fit into the head imaging coil but cannot be used with the standard breast coil and knee coil (Fig. 1a). The current imaging system vendor’s approach to addressing this problem is to use coil-specific phantoms, which are typically liquid-filled bottles or balls that come in various sizes to fit the corresponding RF coils (Supplementary Fig. 1a). However, the simple bottle/ball phantom lacks the capability to characterize some of the most important image quality metrics, such as contrast, spatial resolution, slice thickness, etc. In addition, RF coils have advanced considerably with the development of wireless, flexible, and adaptive coils types in the past decades^19,20^.

The specifications of phantoms need to be upgraded to conform with technological advancement, but a more efficient alternative solution is also needed instead of constantly developing new physical phantoms. Phantoms should allow individually appropriate evaluation for image quality on the anatomical regions of interest, since the concerned imaging characteristics and image quality metrics may vary across those regions. For example, brain MRI is a common examination at many sites. The quality parameters of brain images should be comprehensively evaluated for reliable structural and functional study^21–23^, whereas for breast MRI examinations, fat suppression has been known to be an important tool for detection of pathology. The performance of fat suppression should be specifically evaluated in breast imaging^24,25^. In addition, there is also a growing demand for system phantoms designed to meet specific criteria to help validate quantitative imaging^8^. The structural brain imaging phantom, quantitative breast phantom, and fat fraction phantom have been developed as a system phantom to evaluate certain imaging biomarker reliability^6,26,27^. Consequently, an ideal phantom should be designed for versatility so that it can be easily expanded according to the advances of the MRI system and interchangeably used for multimodal imaging, MRI-based therapy fields, and customized for various RF coils.

To achieve the above-mentioned purpose, we propose a new concept of phantom design using modular architecture, similar to that of a LEGO (Bilund, Denmark) brick. Modularity allows all objects to be subdivided into unit modules with different functions. The unit modules can either be used individually to evaluate certain image quality metric, or be easily assembled using a stud-and-tube coupling system to perform a specific imaging task. Strong interlocking bricks with a geometric tolerance is quite tight, while still allowing the bricks to be easily disassembled. LEGO bricks have been used in previous studies as physical phantoms to evaluate geometric distortion in radiology and radiation therapy^28–31^. However, standard LEGO bricks are limited in their versatility for comprehensive and specific image quality evaluation. For instance, it is impossible to evaluate contrast resolution using contrast generating solution due to the non-closed module structures of LEGO bricks. Also, standard LEGO bricks are not designed to assess image quality parameters such as spatial resolution, slice thickness, and geometric distortion.

The purpose of this study was to develop and validate a LEGO-compatible MOMA phantom with great versatility for general quality assurance and quantitative imaging in MRI. We studied the imaging characteristics of the fabricated individual modules and an assembled MOMA phantom. The reliability for suitable image quality evaluation was verified using a MOMA phantom assembled for head, breast, knee, spine, and body matrix coils.

## Results

### LEGO-compatible MOMA phantom

The developed MOMA phantom was designed in the concept of a “unit module”, which can either be used independently or combined with other units through the modular assembly. Therefore, customized phantoms which are well-suited for the head, breast, and knee RF coils can be easily assembled, as shown in Fig. 1b. There are three types of unit modules: the basic module (BM), the quality module (QM), and the container module (CM). Each unit module has a stud-and-tube coupling system with the strong interlocking function of LEGO bricks. The LEGO bricks can be used as a jig, fixture, and cradle for easily and securely locating the custom assembly phantom inside the coil (Fig. 1c).

### The BM fabrication and imaging characteristics

**Figure 2:**
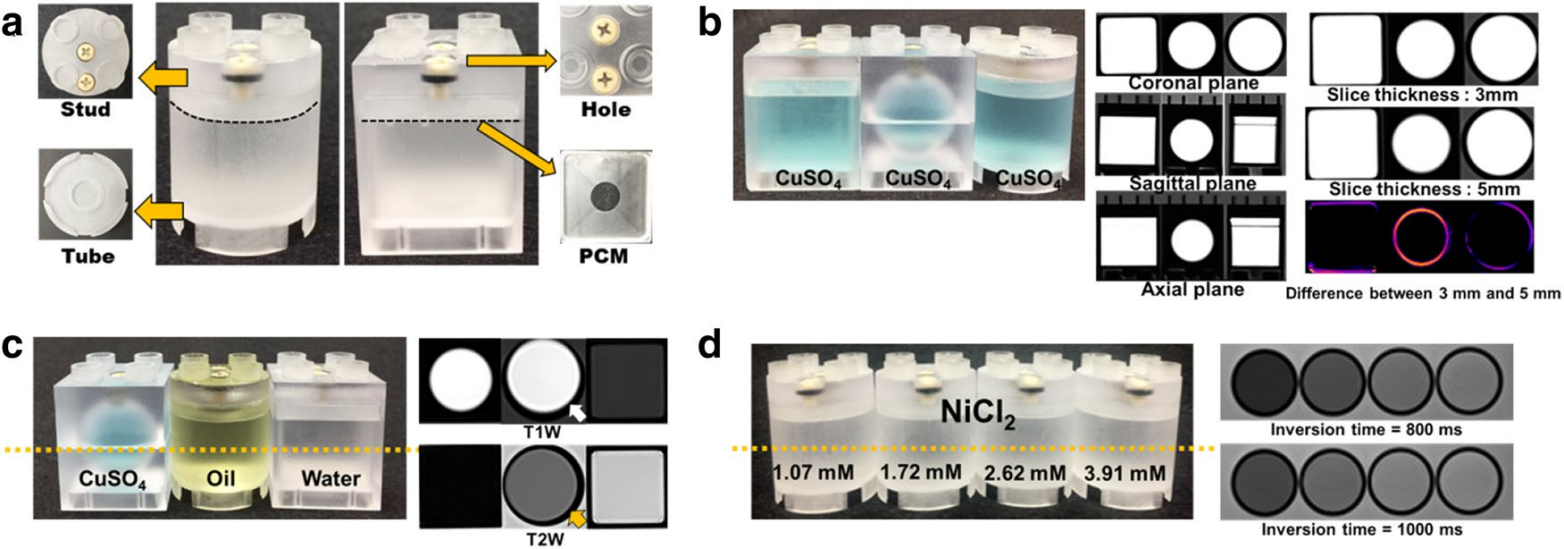
The BM fabrication and imaging characteristics. **(a)** The BM has LEGO-compatible stud-and-tube mechanical couplings and can be easily combined with LEGO products. The BM includes nut holes through which solutions can be injected and sealing rounded head screws that prevent leakage. Air bubbles that generate artifact on images are separated by a thin PCM attached under the stud coupling part. The cylinder and cubic BM have cylinder and cuboid inner structures. **(b)** The cubic, sphere, and cylinder BM containing CuSO_4_ and corresponding T1W images with different slice directions of BMs. The spherical BM images have circular cross-section in any direction. The cylinder and cubic BMs show different cross-section images according to the slice directions. Coronal T1W image with different slice thicknesses. The sphere type BM was more affected by slice thickness parameter than the cubic and cylinder type BM due to the edge curvature in-plane. **(c)** The various inner shapes of BM containing CuSO_4_, oil, and water and corresponding T1W and T2W images. The BM containing CuSO_4_ with the shortest T1 value has high intensity than oil and water BM on T1W images. Water BM with the longest T2 value has high intensity than CuSO_4_ and oil BM on T2W. The white arrow on T1W and the black arrow on T2W shows a chemical shift artifact manifested in the frequency-encoding direction. Image contrast depends not only on the aqueous solution characteristics, but also on the molar concentration of the substance. **(d)** NiCl_2_ solutions with various concentrations of 1.07, 1.72, 2.62, and 3.91 mM were injected in four cylindrical BMs. The axial MR images along the yellow dotted line are shown in the right. The signal intensities inside the BMs were lineally correlated to the NiCl_2_ concentrations.

The BMs are individually enclosed and hollow inside, with a stud-and-tube coupling system for LEGO-compatibility (Fig. 2a). There are bolts and nut holes on one surface of the BMs, which allow users to add specific solutions for desired contrast. The BMs contain a thin polycarbonate membrane (PCM) specially constructed to separate air bubbles from the imaging area. The air bubbles can be moved and trapped in the space between the PCM and the stud coupling part (Supplementary Fig. 2 and Supplementary Note 1). For expandability, the dimensions of the BM were designed to be identical in length and width and twice the height of the traditional LEGO Duplo brick.

The usability and imaging characteristics of BMs were validated by MR images. The central slices of the BMs were used for the imaging feature analysis.

There are three types of inner structures for the BMs containing CuSO_4_: cube, sphere, and cylinder (Fig. 2b). Various inner shapes of the BM show different image characteristics according to the scanning parameters. T1-weighted (T1W) images in the coronal, sagittal, and axial planes of those BMs are shown in Fig. 2b. The spherical BM images have the same cross-sectional shape in all directions, but the cubical and the cylindrical BMs do not. Figure 2b also shows the coronal T1W images with different slice thicknesses of 3 and 5 mm. The coronal images of the cubical and the cylindrical BMs were not affected by the slice thickness. On the other hand, the spherical BM was clearly affected due to the projection of the edge curvature. The specific geometry of the BM inner shape can be chosen according to the study purpose based on the above characteristics.

Representative contrast sources such as CuSO_4_, oil, and water solution were injected into the BMs for the contrast assessment application (Fig. 2c). CuSO_4_, oil, and water have relatively short, intermediate, and long intrinsic T1 values, which resulted in high, intermediate, and low signal intensities on axial T1W images, respectively. At the same time, CuSO_4_, oil, and water have relatively short, intermediate, and long T2 values, which produced low, intermediate, and high signal intensities on axial T2-weighted (T2W) images, respectively. Chemical shift artifact is clearly manifested in the frequency-encoded directions, which are indicated by the white arrow on the T1W image and the black arrow on the T2W image (Fig. 2c).

The BMs can be used to generate a series of image contrasts when filled with solutions made from different concentrations (Fig. 2d). The molar concentration of each BM can be quantified with a certain imaging technique. The BMs were filled with water containing NiCl_2_ at concentrations of 1.07 mM, 1.72 mM, 2.62 mM, and 3.91 mM, as shown in Fig. 2d. The corresponding mean T1 values (± SD) measured by NMR at 22 °C were (1,001.62 ± 14.02) ms, (715.55 ± 10.02) ms, (500.48 ± 7.01) ms, and (353.12 ± 4.94) ms, respectively, using the previously reported method by NIST^32–34^. T1 values calculated from an inversion recovery spin echo method were (984.1 ± 0.3) ms, (706.0 ± 1.5) ms, (496.7 ± 0.4) ms, and (351.5 ± 0.9) ms, respectively. The BM with 3.91 mM NiCl_2_, which has the shortest T1 value, shows the highest intensity on T1W inversion recovery images. The nuclear magnetic resonance (NMR) values can be used as reference values to calibrate quantitative MRI results from various manufacturers’ MRI system.

### The QM fabrication and imaging characteristics

The QMs are units specifically designed to achieve image quality evaluation functions such as spatial resolution, geometric accuracy, and slice thickness accuracy. The dimensions of the QMs could be expanded by multiples of those of the BM unit size according to the inserted test structure size. The QMs also include a stud-and-tube coupling system, bolts and nut holes, and the PCM. The usability of QMs was validated by MRI.

**Figure 3:**
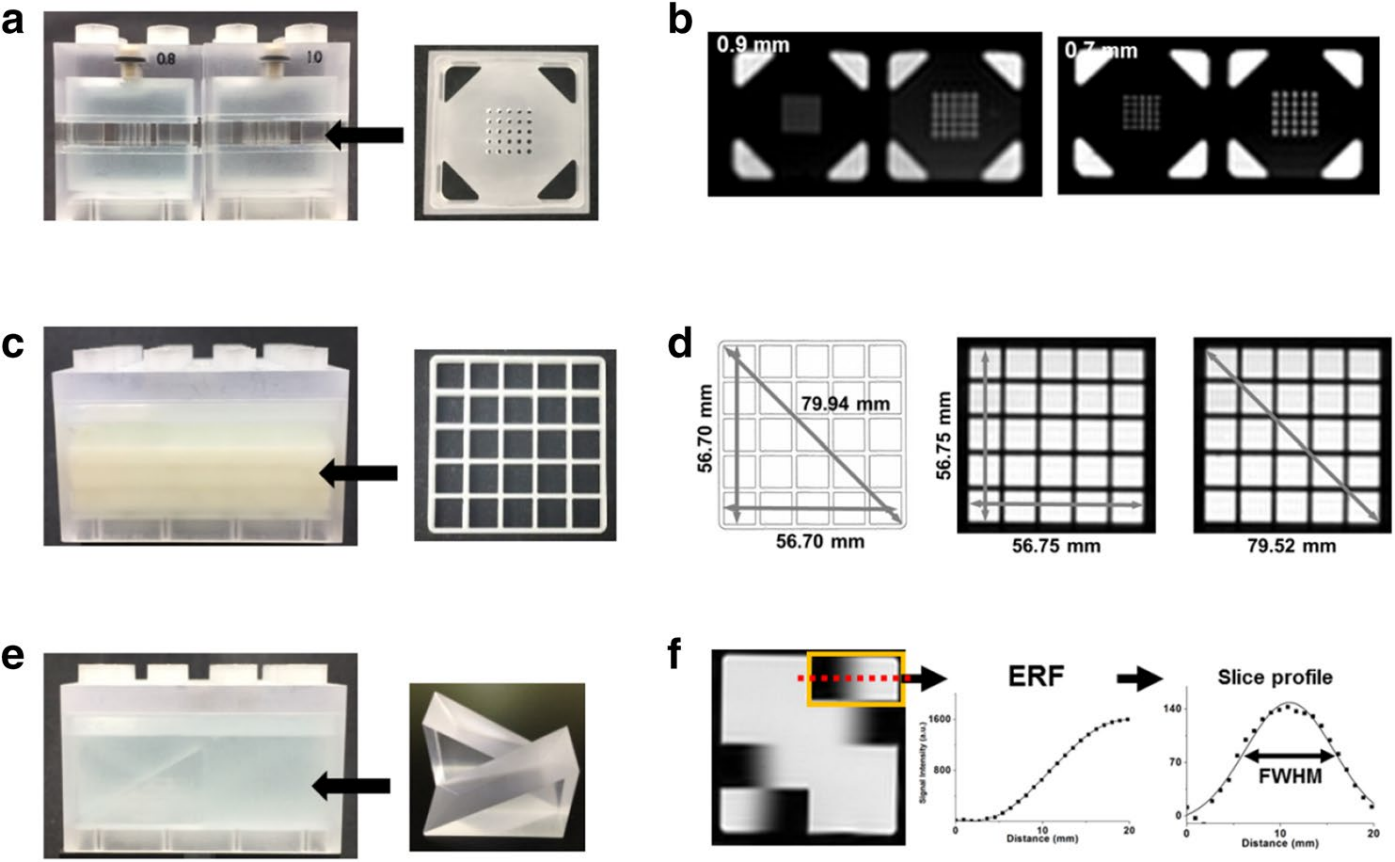
The QM fabrication and imaging characteristics. **(a)** The spatial resolution QMs are constructed from spatial resolution test structures and BM combination. Spatial resolution QMs with 0.8 mm and 1.0 mm holes array and **(b)** the corresponding T1W image with the 0.9 mm and 0.7 mm in-plane resolution of those QMs. The 0.8 mm hole array is sufficiently resolved in the 0.7 mm in-plane resolution image. **(c)** The geometric accuracy QM is constructed from four connected BMs. Grid sheets were inserted as test structures for geometric distortion. **(d)** The physical length of grid sheet which can be imaged on an MRI scanner is 56.70 mm in the vertical and horizontal directions and 79.94 mm in the diagonal direction (gray arrows). The measured corresponding lengths on the image was 56.75 mm along the vertical and horizontal directions and 79.52 mm along the diagonal direction. **(e)** The module size of slice thickness accuracy QM is constructed from quadruple of the BM. **(f)** Pairs of wedges that are designed to evaluate slice thickness were scanned with a slice thickness of 5.0 mm. A line profile was drawn on one wedge image, and the FWHM driven by the derivation of the line profile was calculated as 5.3 mm (red dotted line = line profile on image).

The spatial resolution QM which is designed to evaluate the ability to resolve small objects, has an array of holes of a certain size on the acrylic plastic (Fig. 3a). The spatial resolution of MR images was evaluated by filling the holes with a solution. The 0.8 mm hole array with a 5-by-5 matrix was clearly discernible in a 0.7 mm in-plane spatial resolution T1W image, but was not resolved in a 0.9 mm in-plane spatial resolution T1W image (Fig. 3b).

The geometric accuracy QM, which is designed to evaluate the geometric error, was fabricated by embedding grid sheets (Fig. 3c). The physical inner length of the grid sheet is 56.70 mm in the vertical and horizontal directions and 79.94 mm in the diagonal direction (Fig. 3d). The measured grid lengths on the acquired image was 56.75 mm along the vertical and horizontal directions, which shows a 0.09% difference. In addition, the measured diagonal length of the grid was 79.52 mm, which shows 0.5% difference.

The slice thickness QM contains a pair of opposite wedges with 30° angles attached to the inner base (Fig. 3e). The MR images were acquired with a slice thickness of 5 mm. The derivative of the edge response function (ERF) along the dotted-line profile of the wedge was calculated (Fig. 3f). The full width at half-maximum (FWHM) of the derivative function, which is an estimation of the slice thickness, was measured to be 5.3 mm.

### The CM fabrication and imaging characteristics

The CM is a large solution tank made of acrylic plastic which has filling and drainage ports (Fig. 1c). The inner and the outer basal planes of the CM were made of LEGO-compatible coupling plates, so that the BMs, the QMs, and LEGO bricks can be coupled through these planes. The inner space can be filled with water or other solutions to reduce magnetic susceptibility artifacts from the air/modules interface. The SNR and intensity uniformity can be measured with the CM. The CM can offer relatively large and uniform compartments filled with well-characterized solutions; therefore, it meets NEMA's recommended conditions for measuring those two quantities. The SNR was measured on the image of the CM without other modules (Supplementary Fig. 3).

Appropriate slice positioning is achieved by a slice position indicator accessory module (AM) as shown in Supplementary Fig. 4 and Supplementary Note 2.

**Figure 4:**
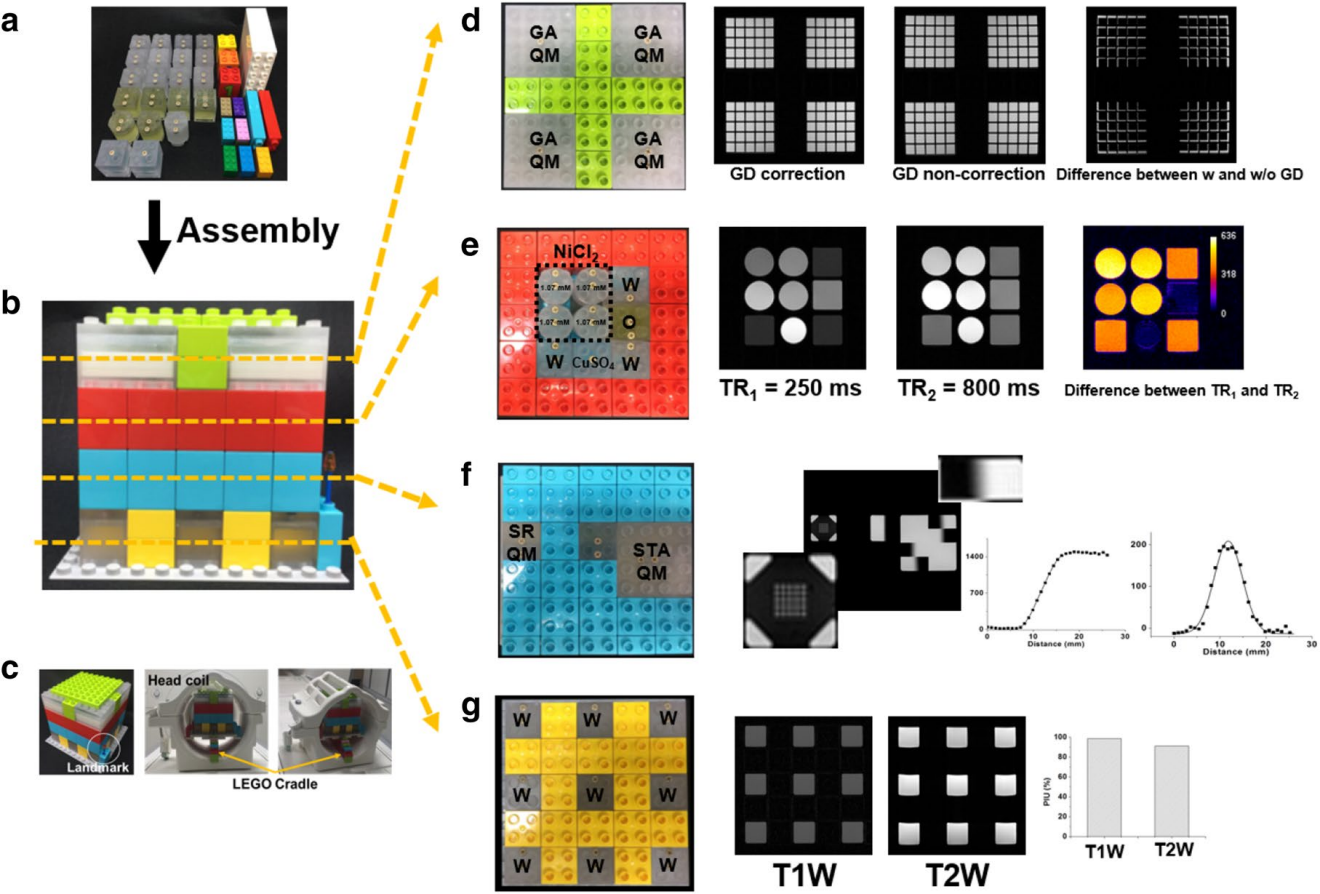
Comprehensive image quality evaluation using customized MOMA phantom assembly for head coil. **(a)** MOMA phantom modules and **(b)** assembled phantom with 4 different sections for individual image quality assessment. **(c)** LEGO bricks were employed as landmarks for phantom positioning and cradle for appropriate positioning. **(d)** Geometric distortion was evaluated by geometric accuracy QMs. Geometric distortion correction option in the scanner operation console was tested. **(e)** Image contrast on different TR was evaluated by 4 cylindrical BMs with NiCl_2_ and 5 BMs that contain water, CuSO_4_, and oil. Different contrasts are clearly depending on the intrinsic T1 value of the associated BMs (Supplementary Fig. 3). **(f)** Spatial resolution and slice thickness accuracy were measured by the corresponding QMs. The QM with a 1.0 mm hole array was resolved on an image with 0.9 mm in-plane resolution. The 3.0 mm of slice thickness was measured as 3.3 mm. **(g)** Intensity uniformity was evaluated using 9 BMs containing water on T1W and T2W images. The PIU on the T1W image was higher than that of the T2W image (*GA* geometric accuracy, *GD* geometric distortion, *SR* spatial resolution, *STA* slice thickness accuracy).

### Customized MOMA phantom assembly for various RF coils

**Figure 5:**
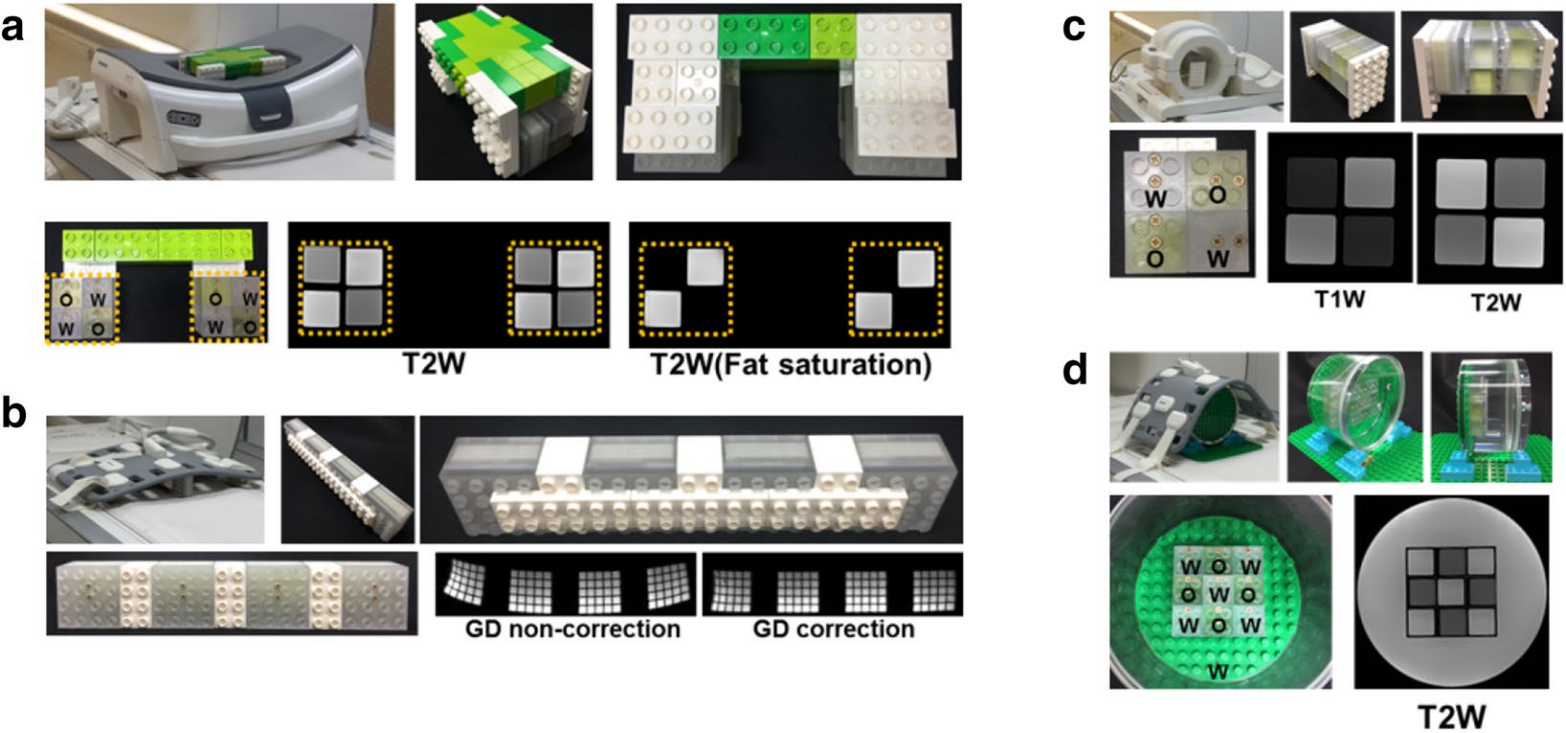
Specific image quality evaluation using MOMA phantom assembly in breast, spine, knee, and body coils. The BM, QM, and CM modules were selected and assembled to test contrast and geometric distortion in various coils. LEGO products were used for module fixture. **(a)** The 2 × 2 matrix of BMs containing oil and water were appropriately assembled in bilateral breast coil configuration. Fat suppression performance was evaluated on T2W images, and BMs with oil material was sufficiently suppressed with the fat saturation option. **(b)** The geometric accuracy QMs with 350 mm length were assembled and imaged with and without geometric distortion correction in the spine coil. **(c)** The T1- and T2-weighted images of assembled 2 × 2 matrix BMs including oil and water in a unilateral knee coil. **(d)** The CM with a 3 × 3 BM matrix, which contains oil and water, was scanned in a body coil (*GD* geometric distortion).

The image quality parameters that are of clinical interest can be very different for different body parts. The MOMA phantom, however, allows for the evaluation of a specific feature for various RF coils including head, breast, spine, knee, and body (Figs. 4, 5).

Image quality parameters such as uniformity, contrast, spatial resolution, slice thickness, and geometric distortion need to be comprehensively evaluated in the region within the 32-channel head coil as illustrated in Fig. 4. MOMA phantom modules (Fig. 4a), including the BMs and QMs were assembled in individual sections and then appropriately positioned using a LEGO cradle which was assembled by LEGO products. LEGO products also were used as landmarks for phantom positioning (Fig. 4b, c). Geometric distortion was evaluated using a 2-by-2 array of 4 geometric accuracy QMs in a geometric section (Fig. 4d). The degree of geometric distortion was affected by the selection of the geometric distortion correction option in the scanner operation console. The barrel-shaped distortion appears without geometric distortion correction. The change in image contrast as a function of the repetition time (TR) was evaluated by 4 cylindrical, 1 spherical, and 4 cubical BMs in a contrast section (Fig. 4e). The cylindrical BMs include different concentrations of NiCl_2_, the spherical BM includes CuSO_4_, and the cubical BMs contain water and oil (Supplementary Fig. 5). Contrast differences can be clearly observed depending on the intrinsic T1 values of each solution. Since the T1 values of the BMs containing oil and CuSO_4_ are short, their signals show similar intensities in images with a different TR. As a result, no signal was observed in the subtracted image. Spatial resolution and slice thickness accuracy was measured by the corresponding QM sections (Fig. 4f). The 1.0 mm holes were successfully distinguished when the in-plane resolution was set to be 0.9 mm. In addition, 3.0 mm of slice thickness was measured as 3.3 mm. The uniformity of pixel intensity was evaluated using 9 BMs containing water on a T1W and T2W image in the uniformity section (Fig. 4g). Identical circular region-of-interest (ROI) were drawn on BM images. The percent integral uniformity (PIU) was 98% and 96% on T1W and T2W images, respectively.

Water and fat separation imaging techniques, including fat suppression, is important in the breast region. The T2W images of BMs containing water and oil were acquired with a breast coil (Fig. 5a). The customized breast coil phantom not only fits in the RF coil easily, but also has the capability to characterize the system performance in terms of fat suppression. Since the spine image field of view is typically long, the image can be easily affected by geometric distortion. The degree of geometric distortion with and without geometric distortion correction was evaluated using serially assembled geometric accuracy QMs with a total length of 350 mm (Fig. 5b). The severe geometric distortion along the superior-inferior direction were remarkably reduced with geometric distortion correction. In addition, the BMs containing water and oil were scanned in the knee coil (Fig. 5c), and the CM combined with BMs containing water and oil was filled with distilled water and scanned in the body matrix coil (Fig. 5d). The corresponding T1W and T2W images of assembled BMs are shown in Fig. 5c, d. This shows that the BMs, QMs, and CMs can be utilized for comprehensive and specific evaluation of image quality among various RF coils.

## Discussion

In this article, we demonstrate the feasibility of a novel MRI image quality evaluation phantom, LEGO-compatible MOMA, which is the customizable medical imaging phantom that can be used for both quality assurance and quantitative imaging. The MOMA phantom enables the evaluation of specific image quality by customizing the desired modules, which can be fit into various MRI coils. Moreover, the proposed MOMA phantom offers great flexibility due to its modular assembly.

With the increasing demands for quantitative imaging, it is essential to develop phantoms that can meet the needs of various clinical applications. The ongoing development of individual phantoms that are isolated and single-functioned is inefficient and costly. However, a comprehensive evaluation of both the qualitative and quantitative features can be achieved through the desired BMs, QMs, and CMs assembly. The BMs can be used to evaluate quantitative responses, such as T1 and T2 relaxation time and proton density measurements by changing the reference materials inside of the modules. The slice thickness, spatial resolution, and geometric distortion can be evaluated by using the QMs with an optimal test structure. Each module provides an independent modularity property. For instance, a spatial resolution QM can be independently used to test the corresponding spatial resolution in arbitrary spatial positions of an MRI scanner, which can be difficult with the traditional phantoms. Furthermore, other image features that were not discussed in this article can also be evaluated by simply adding the optimal test structure into the current modular assembly. The modules used in this study have the basic shape of any module construction which can be extended, similar to wooden toy blocks. Modules can have different sizes and shapes, including circles, curves, and cylinders, but all other module construction will be multiples or fractions of the sizes and shapes of the current modules. This demonstrates the scalability of the MOMA phantom. The compatibility of the MOMA phantom with the LEGO bricks provides new opportunities for phantom usability and versatile expandability. The jig constructed by LEGO bricks can be used as a cradle for accurate and precise positioning of the MOMA phantom for each image acquisition. In the past, LEGO products have been widely used in various imaging modalities and therapy areas such as interventional MRI^35^, four-dimensional computed tomography (4DCT)^36^, radiation therapy tumor tracking^37^, and image guided neurosurgery^38^. The LEGO-compatible MOMA phantom may be easily implemented in the above-mentioned fields with additional functions than LEGO bricks alone.

In this study, three cornerstone modules, BMs, QMs, and CMs, were evaluated in a major pulse sequence, spin echo for T1 and T2. Also, the modules were evaluated in a gradient echo for T1 with short TE. Phantom induced susceptibility artifacts were not observed in those sequences. However, this artifact may be shown in long TE, echo-planar imaging (EPI), and true fast imaging with steady state precession (TrueFISP) sequences. Inevitable air bubbles, air gap created when assembling the BMs and the QMs as well as material borders also may cause susceptibility artifacts especially with large amounts of plastic in the phantom. Extended modules to reduce the susceptibility artifacts will be developed through the paramagnetically doped agarose or similar gels modules. Such modules include the appropriate size and number through magnetic field mapping of modules. This approach is an advantage of phantom modularity.

The coupling system of the modules allows for tight assembly and easy disassembly without significant external forces. Assembly reproducibility between modules can be an important factor in the MOMA phantom approach. Assembly reproducibility was observed by physically measuring five separate BM assembled modules using an optical microscope (Mitutoyo, MF-B2010C). The maximum difference among five separate assemblies from the designed length in the planning drawing was 0.009 mm, which is almost negligible compared to the limiting spatial resolution (0.5 mm) of the MRI system. Another limitation is the user-fillable structure of the modules, which make it challenging to maintain consistency of the well-characterized filling material. One option to address this issue is to completely seal the modules after pre-filling of the desired critical solutions, which need to maintain consistent and traceable characteristics. In this case, the holes would be sealed by an ultrasonic plastic welding technique that prevents leakages and filling. Finally, in terms of cost, the MOMA phantom is expected to have similar costs as the existing phantoms. However, the maintenance cost is expected to be much lower than other phantoms, because the MOMA phantom is easily upgradeable by adding individual modules.

The results discussed in this paper suggest that the MOMA phantom can provide suitable image quality evaluation through customization, and efficient phantom management through a modular approach. In addition, LEGO-compatibility consistently allows usability and expandability of phantom applications in various MRI systems. Further research should focus on improving the MOMA phantom to create a standard phantom package for quantitative imaging by including traceable components, the standard imaging protocols, the image analysis procedures, and the building instructions to ensure the stability of the module components^8^.

## Methods

### Module fabrication

The BMs were fabricated in cubical unit bricks with dimensions of 31.70 mm × 31.70 mm × 43.00 mm, and in cylindrical unit bricks with an inner diameter of 29.70 mm and a height of 43.00 mm. The cubical BMs have two types of inner shapes, which are the cuboid form with inner dimensions of 29.70 mm × 29.70 mm × 24.40 mm and the spherical form with a 25.0 mm inner diameter. The BMs were made of PC using computer numerical control (CNC) machining. The PC was selected because of its durability, semi-transparency, and stability over a greater temperature range. The density of PC is 1.2 g/cm^3^ as listed in the American Society for Testing and Materials (ASTM) D792, and its water absorption (ASTM D570) is 0.15%. The BMs were composed into two parts: the upper part with the stud portion and the lower part with the tube portion, which were welded together using an ultrasonic plastic welding technique. The 1.0 mm hole and 0.2 mm thickness of the PC membrane was embedded to separate air bubbles in the BMs.

The QM test structures were made of PC, PMMA, and polyoxymethylene (POM) using CNC drilling according to the test structure characteristics. The density of PMMA (ASTM D792) and POM (ASTM D792) were 1.19 g/cm^3^ and 1.41 g/cm^3^, respectively. The QMs consist of three parts: the BM stud, the image quality test structure, and the BM tube. Typically, an image quality test structure is glued to the inside of the tube section, which is then welded together with the stud using ultrasonic plastic welding techniques. The PC membrane was also embedded in the QMs to separate air bubbles from the imaging region of the modules. The embedded test structures include representative image quality evaluation parameters, such as spatial resolution, slice thickness accuracy, and geometric accuracy. Spatial resolution test structures with different hole sizes can be used to test the ability to resolve small objects. These were made of PMMA, which was selected because it offers a smoother edge during machining compared to PC. Hole diameters were chosen to be 1.0 and 0.8 mm and the space between each hole was the same as the diameter of the hole. The depth of the hole was 10.0 mm. Slice thickness accuracy test structures contain pairs of opposed wedges. These were made of PC, which was attached to the inner base that is 4 times bigger than that of the cubical BMs. The angle of the wedge is 30°. Geometric accuracy test structures were made of POM, which was also attached to the inner base that is four times bigger than that of the cubical BMs. Differences between predefined physical lengths and measured lengths on grid images is defined as geometrical distortion for MRI. The physical length of the POM grid sheet is 59.70 mm × 59.70 mm. The wall thickness and interspace between the square is 1.5 mm, and the inner length of the square is 10.14 mm × 10.14 mm. As a result, the physical length of the grid sheet which will be imaged on MRI is 56.70 mm.

The CM was made of PMMA. It has a cylindrical shape with openings at the top surface which can be used to fill the phantom with the desired solution. The outer diameter of the CM was 150.0 mm, and the height was 120.0 mm. The size of the CM can be easily adjusted according to the specific requirement. The wall thickness was 5.0 mm to prevent water leakage from physical cracks.

### Evaluation of image quality parameters

To calculate T1 values in the BMs, signal from the inversion recovery spin echo (IR-SE) images were extracted and calculated as follows:

1 $$S\left(TI\right)={A-Be}^{-TI/T1}$$

where TI is the multiple inversion times, $S\left(TI\right)$ is the pixel intensity at TIs. The value of A and B were estimated by the nonlinear least-square curve fitting model.

Spatial resolution was evaluated by visual assessment of the individual small bright spots on the spatial resolution QM modules images.

Slice thickness accuracy was determined by measuring a line profile across the wedges. The derivative of the line profile indicates the scaled slice profile that was projected at the angle of wedge by the true slice thickness. The slice thickness for MRI was defined as follows:

2 $$\text{Slice thickness}=\mathrm{tan}\theta \times FWHM$$

where the full width at half maximum (FWHM) was obtained from the Gaussian fitting of the scaled slice profile.

In order to evaluate geometric accuracy, the total lengths of the grid along both phase and frequency encoding directions were measured and compared to the known size of the grid. The amount of geometric distortion was evaluated based on the difference between the known and the measured lengths.

The uniformity of the image intensity was measured on the BM images. Percent integral uniformity (PIU) between modules was defined as follows:

3 $$\text{PIU (}\%)=\left(1-\frac{{\text{Max}}-{\text{Min}}}{{\text{Max}}+{\text{Min}}}\right)\times 100$$

where Max and Min are highest and lowest intensities among the mean pixel values of a circular ROI on the BM images, respectively. High PIU values indicate good uniformity in signal intensities across the whole image.

### Image acquisition

All image acquisitions were carried out on a 3 T MRI scanner (MAGNETOM Skyra, Siemens healthcare) using various RF coils. The BMs and QMs were scanned for test imaging characteristics in a 32-channel head coil. The 2D spin echo T1W images were acquired with the following imaging parameters: TR = 480 and 800 ms, TE = 25 ms, matrix size = 256 × 256, FOV = 250 × 250 mm^2^, and slice thickness = 5 mm. The 2D fast spin echo T2W axial images were acquired with the following imaging parameters: TR = 2,800 ms, TE = 40 and 80 ms, matrix size = 256 × 256, FOV = 250 × 250 mm^2^, and slice thickness = 5 mm. T1 measurements from the NiCl_2_ BMs were acquired using IR-SE protocols with seven different inversion times (TIs) of 21 ms, 100 ms, 200 ms, 400 ms, 800 ms, 1,600 ms, and 3,200 ms and a fixed repetition time (TR) of 10,000 ms. Imaging parameters on head, breast, spine, knee, and body imaging coil for comprehensive and specific evaluation of the BM, QM, and CM usability is summarized in Supplementary Table 1.

## Supplementary information

Supplementary Information.
