# Supplementary material for: LEGO-compatible modular mapping phantom for magnetic resonance imaging
Source: Sci Rep. 2020 Sep 8;10:14755. doi: 10.1038/s41598-020-71279-1 (PMC7478958; doi:10.1038/s41598-020-71279-1)
Supplement: Supplementary file 1 — Supplementary Information. [file 41598_2020_71279_MOESM1_ESM.docx]

**LEGO-compatible Modular Mapping Phantom for Magnetic Resonance Imaging**

**Hyo-Min Cho^1^, Cheolpyo Hong^2^, Changwoo Lee^1^, Huanjun Ding^3^, Taeho Kim^4^, Bong-young Ahn^1*^.**

^1^Center for Medical Convergence Metrology, Korea Research Institute of Standards and Science (KRISS), Daejeon 34113, Rep. of Korea.

^2^Department of Radiological Science, Daegu Catholic University, Gyeongsan-si, Gyeongbuk, 38430, Rep. of Korea

^3^Department of Radiological Sciences, University of California, Irvine, CA 92697, USA

^4^Department of Radiation Oncology, Washington University, Saint Louis, MO 63110, USA


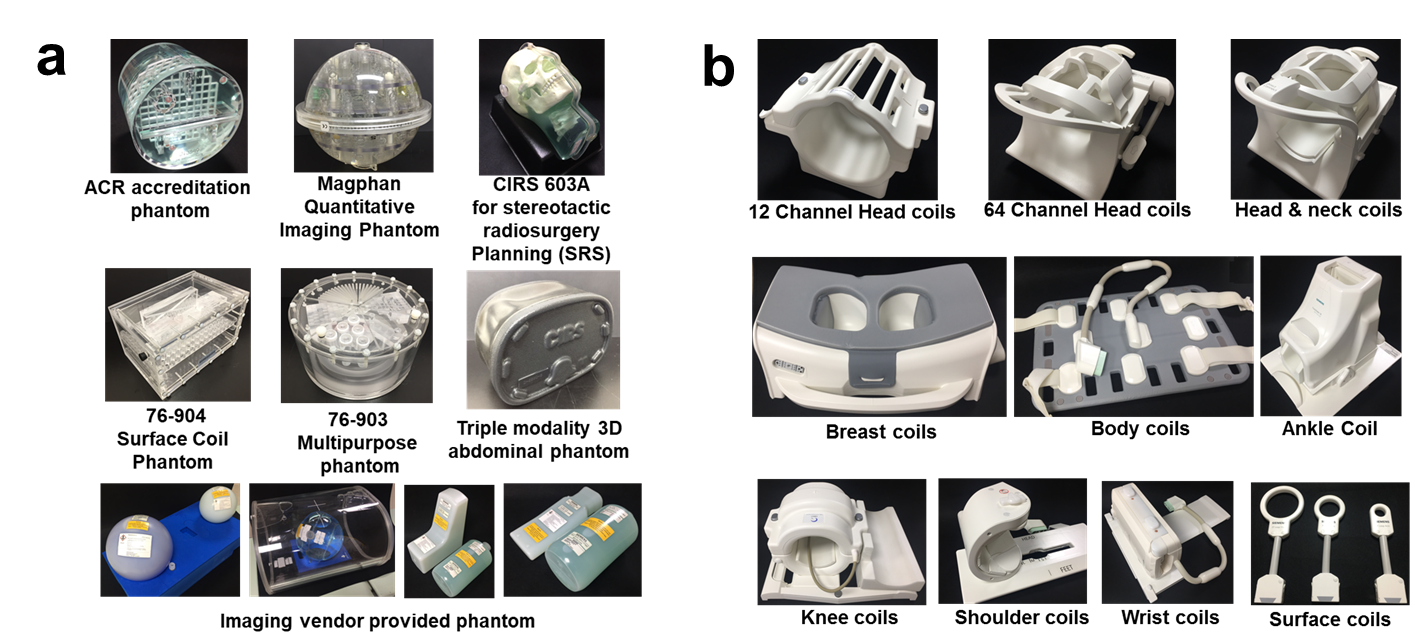


Supplementary Figure 1. (a) Representative physical phantoms for image quality assessments from phantom manufacturers, imaging system vendors and international organizations on MRI. (b) Typical RF coils to cover the region of interest, such as head, breast, body, knee, wrist, and shoulder.


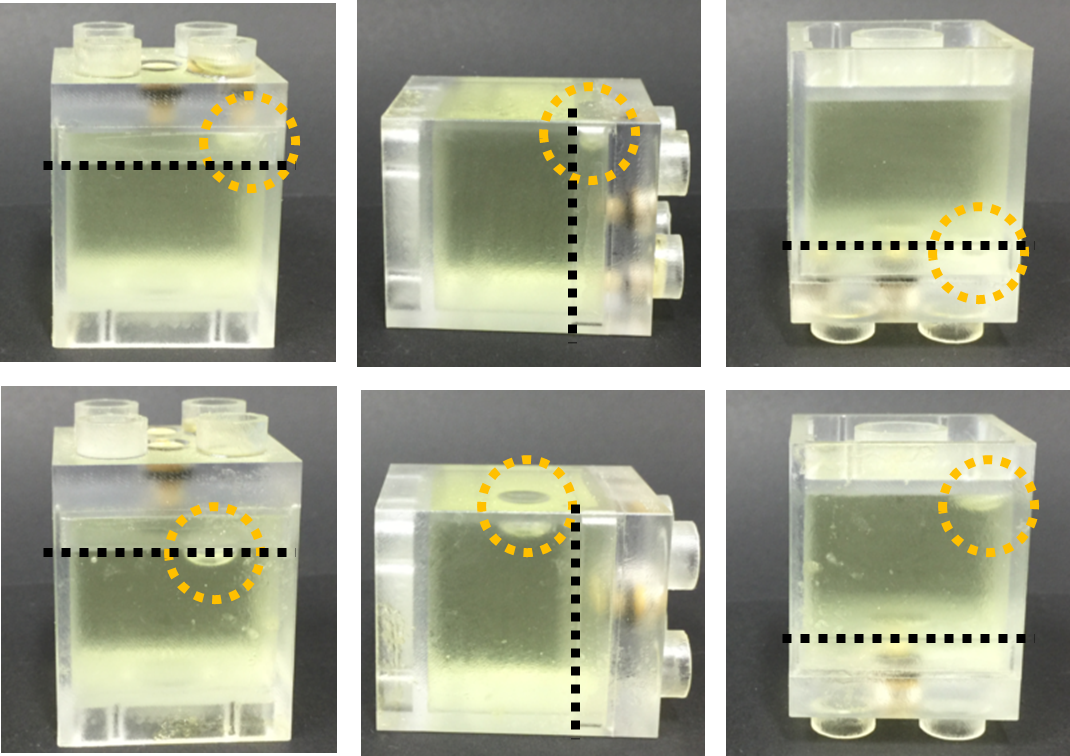


Supplementary Figure 2. The air bubble (yellow circles) according to phantom positions. The upper line shows the air bubble is trapped in PCM (dotted lines). The bottom line shows the non-trapped bubble moves arbitrarily.


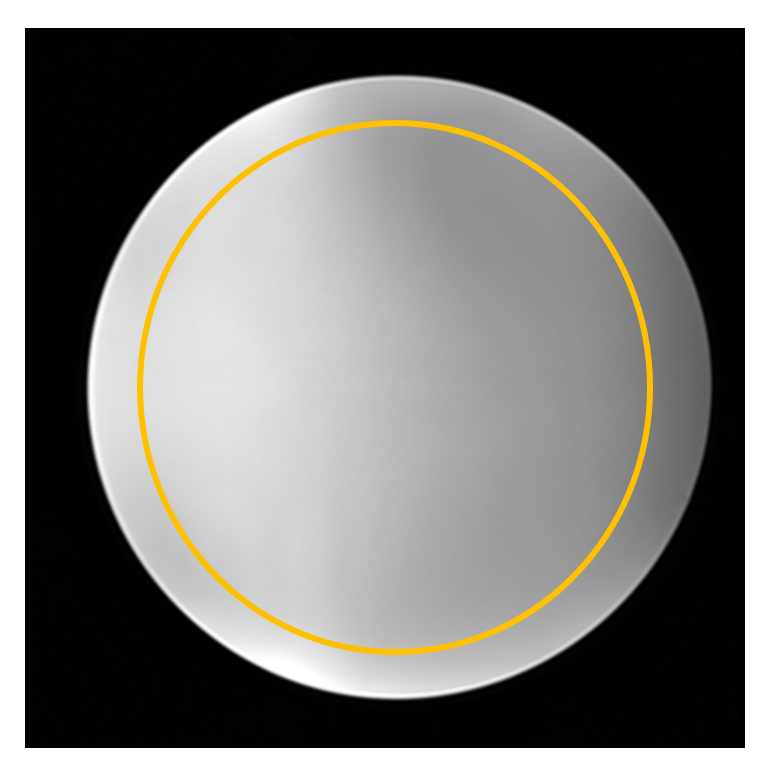


Supplementary Figure 3. The signal-to-noise ratio (SNR) evaluation using a container module (CM). The SNR was calculated by the subtraction method. The two identical images were acquired and subtracted. An ROI (yellow circle) of the CM image was used to the mean signal value. Noise was the standard deviation of the pixel values within the identical ROI. The factor of $\sqrt{2}$ was applied to prevent error proportion of difference image for the SNR calculation. The calculated SNR value was 971.0.


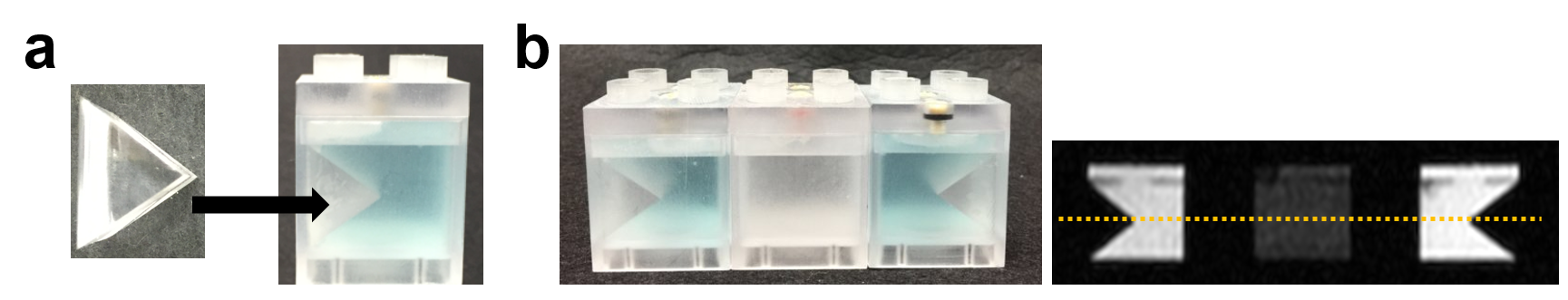


Supplementary Figure 4. The slice position indicator accessory module (AM) (a) Photograph of the wedge and the inserted wedge inside the BM (b) The slice position indicator for the AM and BM and the corresponding module images on a localizer image (yellow dotted line= selected slice position)


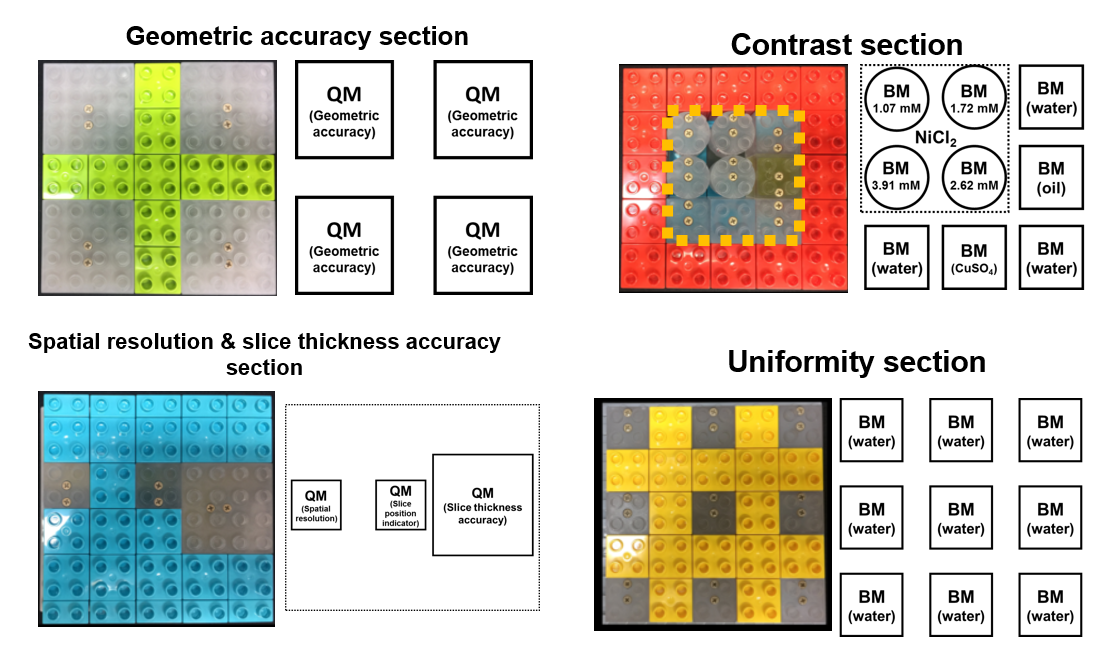


Supplementary Figure 5. LEMON modules arrangement in individual sections on the head coil

Supplementary Table 1: scanning protocols

|  | Sequence | TR  (ms) | TE  (ms) | FOV | Matrix | Slice Thickness(mm) |
| --- | --- | --- | --- | --- | --- | --- |
| Brain | GRE T1W  TSE T2W | 250  6500 | 2.1  97 | 230ⅹ230  230ⅹ230 | 256ⅹ256  256ⅹ256 | 5  3 |
| Breast | TSE T2W  T2W fat sat | 3000  4690 | 66  66 | 280ⅹ280  280ⅹ280 | 384ⅹ384  384ⅹ384 | 3  3 |
| Spine | 3D FLASH (Fast low angle shot) | 7.7 | 3.6 | 420ⅹ280 | 288ⅹ256 | 6 |
| Knee | SE T1W  TSE T2W | 550  4630 | 11  61 | 150ⅹ150  150ⅹ150 | 384ⅹ384  384ⅹ384 | 4  4 |
| Body | TSE T2W | 6750 | 104 | 256ⅹ256 | 256ⅹ256 | 3 |

**Supplementary Note 1. PCM**

The PCM is a septum that has a small circle in the center. The probability of the trapped air bubbles in PCM move through a small hole is very low. However, if the size of the air bubble is too large to move above the PCM, the solution should be re-injected. We had monitored module to check whether air bubbles stay above the PCM after move air bubbles above the PCM. Bubbles were stay well above the PCM unless shaking the module on purpose.

**Supplementary Note 2. Appropriate slice selection using an AM on a localizer image**

Appropriate slice positioning is achieved by a slice position indicator accessory module (AM). The slice indicator AM was constructed by adding a plastic wedge inside the BM.

The slice indicator AM was utilized in a scan scout image for appropriate slice position selection. The slice was positioned on the straight line between wedge vertexes on both sides. This geometry was easily distinguished from other modules and can be used to conduct a reproducible scan.
